# Determinants of undernutrition among adult patients receiving antiretroviral therapy at Debre Markos referral hospital, Northwest Ethiopia: a case-control study design

**DOI:** 10.1186/s40795-019-0284-9

**Published:** 2019-03-01

**Authors:** Ayenew Negessie, Dube Jara, Mekaunint Taddele, Sahai Burrowes

**Affiliations:** 1 Department of Nutrition and Food Sciences, College of Medicine and Health Science, Debre Markos University, P.O. Box 269, Debre Markos, Ethiopia; 2 Department of Public Health, College of Medicine and Health Sciences, Debre Markos University, P.O. Box 269, Debre Markos, Ethiopia; 3 Public Health Program, College of Education and Health Sciences, Touro University California, 1310 Club Drive, Mare Island, Vallejo, CA 94592 USA

**Keywords:** HIV/AIDS, Antiretroviral treatment, Ethiopia, Malnutrition, Body mass index

## Abstract

****Background**:**

A complex and negatively reinforcing relationship exists between infection with Human Immune Deficiency Virus (HIV) and malnutrition. HIV-induced immune impairment and its resulting opportunistic infections (OIs) can lead to malnutrition and nutritional deficits, can, in turn, hasten the progression of HIV infection and reduce chances of survival. The determinants of undernutrition among patients receiving antiretroviral therapy (ART) is poorly understood in Ethiopia, despite a high prevalence of food-insecurity that overlaps with a generalized HIV/AIDS epidemic. Therefore, this study aimed to assess determinants of undernutrition among adult patients receiving antiretroviral therapy at Debre Markos Referral Hospital in Northwest Ethiopia.

**Methods:**

We conducted an institution-based, unmatched, case-control study with 636 adult patients receiving antiretroviral therapy. We randomly selected 212 patients with poor nutritional outcomes (cases) and 424 without undernutrition (controls) and then conducted a chart review to collect information on their treatment, socio-economic, and demographic background. Data were analyzed using bivariable and multivariable logistic regression to identify factors associated with under nutrition.

**Results:**

We found that greater age (AOR = 1.02, 95% CI: 1.01,1.05), fair or poor adherence (AOR = 2.77, 95% CI: 1.40, 5.50 and AOR = 4.72, 95% CI: 1.92, 11.6), and the presence of OIs (AOR = 1.70, 95% CI: 1.12, 2.52), anemia (AOR = 1.81, 95% CI: 1.07, 3.07), or eating problems (AOR = 3.40, 95% CI: 2.27, 5.10), were all independently and positively associated with under nutrition. Starting treatment with a medium or low CD4 count was protective (AOR = 0.61, 95% CI: 0.39, 0.96 and AOR = 0.49, 95% CI: 0.27, 0.88). Having social support (AOR = 0.64, 95% CI: 0.43, 0.95), and having a source of informal care-giving (AOR = 0.48, 95% CI: 0.27, 0.84), reduced the odds of undernutrition.

**Conclusion:**

Our findings support calls for treating HIV infection early and aggressively, while closely monitoring patients for opportunistic infections that might affect eating and drug side effects that may affect appetite. The role of disclosure, peer-caregivers and age in preventing undernutrition should be explored in future research.

## Background

A complex and negatively reinforcing relationship exists between infection with Human Immune Deficiency Virus (HIV) and malnutrition. HIV-induced immune impairment and its resulting opportunistic infections (OIs) can decrease food intake; increase energy expenditure; cause nutrient malabsorption; and alter the body’s ability to utilize and excrete nutrients leading to nutritional deficiencies and poor overall nutritional status [1]. Nutritional deficits, can, in turn, hasten the progression of HIV infection and increase the risk of developing opportunistic infections (OIs). This, in turn, affects overall clinical outcomes, quality of life, and chances of survival [2–6]. In addition, recovering from malnutrition is difficult among individuals with HIV infection. HIV-infected patients, particularly those who are wasted, require greater protein and micronutrient intake than those who are not HIV-infected to support and improve a weakened immune system [7]. While malnutrition is common among patients with advanced, untreated, HIV-infection [3] individuals are at risk of developing undernutrition at every stage of HIV infection [8]. In communities with high HIV prevalence the negative synergies between undernutrition and HIV-infection can impact community-level and national-level health statistics [9].

Antiretroviral therapy (ART) for HIV-infected patients was thought to be a solution to HIV-associated wasting and malnutrition. It is well established that a majority of clinically malnourished people living with HIV who initiate ART will increase or stabilize their weight, even without therapeutic or supplementary feeding support [10]. However; malnutrition remains a problem for individuals on ART [11]. Patients who initiate ART with an undernutrition may have poorer outcomes than their well-nourished counterparts. Furthermore, being well-nourished has been shown to positively affect ART adherence [12–16]. Lack of food, food insecurity, and the worry about food has been shown to reduce drug adherence [7, 17]. In addition, food, ART, and medications for opportunistic infections may interact in ways that reduce the efficacy of ART; and ART itself might impact nutrient absorption, metabolism, and excretion as well as causing side effects that reduce appetite [1].

Continued study of the relationship between malnutrition and ART is particularly important in low-income countries such as Ethiopia where high levels of food insecurity and poverty overlap with generalized HIV epidemics [2, 18, 19]. Food insecurity in these settings leads to widespread, chronic malnutrition, which may complicate treatment and compound HIV-related health problems. Ethiopia has been relatively successful at keeping its adult HIV prevalence to 1.5%, but this figure represents a large number of people: 793,700 people living with HIV including 200,300 children [20]. It is estimated that 420,000 Ethiopians (59% of those in need) are currently receiving ART [21]. Approximately 9% percent of Ethiopia’s population is severely malnourished and the country’s rate of stunting is 38% [22, 23]. It is, therefore, reasonable to expect that many Ethiopians receiving ART struggle with obtaining a diet of adequate quality and quantity. The few studies that have been conducted on this topic in Ethiopia, have found a high prevalence of malnutrition among ART patients [24–27].

Assuring adequate access food in HIV treatment programs has been a central demand of people living with HIV (PLHIV) in low-income countries and, as such, nutritional counseling and therapeutic feeding have been incorporated into HIV treatment programs and tested for efficacy [28–30].

However, our understanding of how undernutrition and HIV treatment interact, and specifically what factors influence individuals’ risk of malnutrition while on ART, for sub-Saharan African counties, is still spotty with a relatively few studies conducted given the large overlap between food insecurity, malnutrition, and HIV-infection in many of these countries. Only a handful of studies on this topic have been conducted in Ethiopia.

This study adds to the knowledge base on this topic by assessing the determinants of undernutrition among adult patients receiving antiretroviral therapy at Debre Markos Referral Hospital, a large tertiary hospital in Ethiopia with an established HIV treatment program.

## Methods

### Study area and setting

This study was conducted at Debre Markos Referral Hospital, in Debre Markos Town, which is located 299 km northwest of the capital city, Addis Ababa. The hospital has a single ART clinic that serves more than 8000 pre-ART and ART clients. The clinic’s patient flow is approximately 1000 monthly with approximately 30 patients daily. At the time of the study, there were 8995 total registered patients at the clinic, of which 3643 were on ART. Of the registered ART patients; 150 were pregnant 38 had been lost to follow up, 271 had died, and 1152 had not been in touch with the clinic for more than three months. The remaining 2022 patients were receiving ART and eligible for the study. Of these, 1700 were over the age of 18 [31]. This was our sampling frame. The study was conducted in 2016 using patient data from September 11, 2008 to August 11, 2015.

### Study design and population

This is an institution-based, unmatched, retrospective case-control study. All adult patients on ART who were registered at the Debre Markos Referral Hospital ART clinic between September 11, 2008 to August 11, 2015, and who had complete registration cards showing current follow-up were eligible for inclusion. Cases were patients who had a record of undernutrition in 2015. We define undernutrition as having a BMI < 18.5 kg/m^2^. Controls were patients with normal nutritional status (BMI between 18.5 Kg/m2 and 24.9 kg/m^2^) in 2015. Patients whose patient cards had incomplete data, pregnant women, and patients who had been transferred from other health institutions were excluded from the study for the sake of data quality.

### Sample size and sampling techniques

We calculated our sample size with EpiInfo (version 7), using double-population proportion formula. Our outcome of interest was undernutrition at any endpoint specific for each patient during the study period. We estimated the proportion of the patient population that would be malnourished by reviewing patient records to find how many patients had reported problems with eating, as eating problems was found to be the main determinant of undernutrition in a study population comparable to ours at Gondar Referral Hospital in Ethiopia [32]. In that study, the proportion of patients with eating problems was 15.3% and the adjusted odds ratio for the association between undernutrition and eating problems was 2.35. Using this information, a two-to-one (2:1) allocation ratio of control to a case, a 5% level of significance (two-sided), and a power of 80%, we calculated a total required sample size of 636 patients with 212 cases and 424 controls.

We selected our sample by extracting a list of all of the eligible patients on ART in the clinic from the clinic registration book. This list was divided into cases and controls based on the patient BMIs recorded in the registration book. We then used simple random sampling to select a random sample of participants from the case and control lists using SPSS (version 20).

### Study variables and measurement

The dependent variable of the study is undernutrition at any endpoint specific for each patient during the study period. This a dichotomous variable, coded as 1 if the patient had a BMI < 18.5 kg/m^2^ and 0 if the patient had a BMI between 18.5 and 24.9 kg/m^2^ based on the World Health Organization’s (WHO) 2006 BMI classification scale [33].

We collected and reported four categories of independent variables (see Table 1). The first category included standard socio-demographic information such as gender, age, marital status, whether the patient had any children and how many, religion, ethnicity and urban or rural residence.

**Table 1:** Sample characteristics among adult patients on ART at Debre Markos Referral Hospital, Northwest Ethiopia, from September 11, 2008 to August 11, 2015 (*n* = 212 cases and 424 controls)

Patient Characteristics	Response	Cases (%)	Controls (%)
Socio-Demographic Characteristics			
Sex	Male	75 (35)	160 (38)
	Female	137 (65)	264 (62)
Age category	18–35 years	118 (56)	272 (64)
	36–55 years	87 (41)	136 (32)
	> = 56 years	7 (3)	16 (4)
Religion	Orthodox	207 (98)	409 (96)
	Other	5 (2)	15 (4)
Marital status	Married	96 (45)	220 (52)
	Single	25 (12)	53 (12)
	Divorced	54 (25)	100 (24)
	Widowed	37 (17)	51 (12)
Ethnicity	Amhara	205 (97)	416 (98)
	Others	7 (3)	8 (2)
Residence	Rural	39 (18)	57 (13)
	Urban	173 (82)	367 (87)
Educational status	Primary school & lower	149 (70)	283 (67)
	Secondary & higher	63 (28)	141(33)
Had children	Yes	137 (65)	289 (68)
Socio-Economic Characteristics			
Has source of external support	Yes	169 (80)	385 (91)
Has disclosed HIV/AIDS status	Yes	82 (39)	257 (61)
Have public services	Yes	150 (71)	338 (80)
Owns a phone	Yes	95 (45)	256 (61)
Piped water	Yes	104 (69)	184 (54)
Own home	Yes	147 (98)	332 (98)
Electric light	Yes	96 (64)	262 (78)
Clinical Characteristics			
Baseline CD4 count	< 200 cells/ul	142 (67)	216 (51)
	200–350 cells/ul	47 (22)	131 (31)
	> 350 cells/ul	23 (11)	77 (18)
Current CD4 count	< 200 cells/ul	24 (11)	52 (12)
	200–350 cells/ul	59 (28)	79 (19)
	> 350 cells/ul	129 (61)	293 (69)
WHO clinical stage at baseline	Stage I	33 (16)	90 (21)
	Stage II	26 (12)	91 (21)
	Stage III	132 (62)	215 (51)
	Stage IV	21 (10)	28 (7)
Current WHO clinical stage	Stage I	209 (99)	422 (98)
	> Stage I	3 (1)	2 (2)
ART adherence	Good	161 (76)	394 (93)
	Fair	28 (13)	21 (5)
	Poor	23 (11)	9 (2)
Took therapeutic food	Yes	59 (28)	4 (0.94)
Had eating problems	Yes	137 (65)	112 (26)
Had opportunistic infection	Yes	124 (58)	143 (34)
Had anemia	Yes	44 (21)	48 (11)
Total duration of ART follow-up	<= 12 months	7 (3)	26 (6)
	> 12 months	205 (97)	398 (94)
Follow-up interval	<= 3 months	209 (99)	420 (99)
	> = 3 months	3 (1)	4 (1)
Pre-ART & ART follow up duration	<=12 months	5 (2.4)	18 (4)
	> 12 months	207 (97.6)	406 (96)

The second batch included proxies for household income and wealth. This includes the data on whether the patient had their own home, the number of rooms and residents in the home, whether the patient had access to piped water and electricity and whether the patient owned a mobile phone.

The third batch included patient treatment history and clinical characteristics that might be related to undernutrition. Included were the patient’s total duration on ART; WHO clinical stage at intake; whether during the course of care, the patient had experienced any malignancies and type of malignancy; whether the patient had experienced any opportunistic infections (OI) and type of infection; whether the patient had been anemic (measured by hemoglobin level); whether the patient had experienced eating problems or received therapeutic food and the cause of eating problems; the patient’s baseline CD4 count; the patient’s current CD4 count at the time of the study; ART adherence; and the total duration of pre-ART and ART follow up. Patients were said to have had an opportunistic infection, if they developed at least one of opportunistic infections listed in Harrison’s *Principles of Internal Medicine* [34].

The adherence to ART was assessed using percentages and was calculated using number of doses missed for the last one month. Adherence percentage equal to or greater than 95% or ≤ 3 doses missed per month is considered as Good, 85–94% or 4–8 doses missed per month considered as Fair, and less than 85% or ≥ 9 doses missed per month is considered as Poor [35]. Finally, we collected data on whether the patient had disclosed their HIV status and to whom they had disclosed; and whether they had personal source of psychosocial support and the source of support (parents, sisters, brothers, relatives, wife or husband, or priest).

### Data collection methods

Our main data source was the ART clinic registration book and the patient’s registration card. We checked the completeness of the data in the ART clinic’s registration book before beginning data extraction. We developed a data extraction checklist based on the variables we wanted to include in the study and conducted a pre-test on random sample of patient registration cards (equal to 10% of our sample size), to ensure its consistency and completeness.

The data collection process was overseen by the project’s principal investigator. We recruited a team of two public health professionals and a BSc nurse who were not associated with the ART clinic as data collectors. They were given a one-day training on the objectives of the study, the method of data collection and how to extract information from patient registration cards and the registration book using the checklist. We also communicated with individuals from Hospital card office and briefly trained them about the study process and the requirements.

Hospital health informatics technicians assisted us in tracing clinic patients’ hospital registration cards (i.e.*,* their medical records) using the patient’s registration codes. Each patient/registration card was then assigned a unique anonymous identification number, which was recorded on our data extraction checklist. We extracted data from patient registration cards and from the registration book, using the pre-tested checklist. The collected data were entered in EpiData (version 3.1) to minimize data entry error and then exported to STATA (version 14) for analysis.

### Data analysis

We calculated the frequency and percentage distribution of the variables under consideration and then fitted a logistic regression model identifying the factors associated with undernutrition. We included in our multivariable model: a standard battery of demographic control variables, variables that were found to be associated with undernutrition in bivariable regressions at the 20% level [36], and variables that have been found previous studies to be associated with undernutrition and poor clinical outcomes in those receiving ART. Variables that met the criteria above but that had small exposures and created sparse cells were also omitted [36]. The crude and adjusted odds ratios together with their corresponding standard errors were computed. A *p*-value less than or equal to 0.05 was considered statistically significant.

## Results

### Demographic characteristics

A total of 636 patients were studied for this project (212 cases and 424 controls). The study sample was disproportionately female (63% in total--65% for cases and 62% for controls) and most study subjects were between the ages of 18–35 (see Table 1). The vast majority of patients belonged to the Amhara ethnic group and were Ethiopian Orthodox Christians (> 90% in both cases). The plurality of the sample was married (45% of cases and 52% of controls) and most had children (> 60% for both cases and controls). Approximately 14% were widowed.

The sample was predominantly urban (82% of cases and 87% of controls) and relatively well-educated: 32% had a secondary school education or higher. And, reflecting their urban residence, the majority of the sample had access to public municipal services (71% of cases and 80% of controls) and a large proportion, relative to the Ethiopian average, had access to piped water (69% of cases and 54% of controls), electricity (64% of cases and 78% of controls), and a phone (45% of cases and 61% of controls). Almost all respondents (> 98%) owned their own home (Table 1).

### Clinical characteristics

The average amount of time that patients had been receiving care at the clinic was 5.7 years (±2.1SD) for cases and 5.6 years (±2.4SD) for controls. Most patients started ART with advanced disease: 67% of cases and 51% of controls had a baseline CD4 count < 200 cells/ul before starting ART; and 72% of cases and 57% of controls started treatment at WHO stage 3 of disease or higher. Our sample of patients had been receiving ART for 5.2 years (±1.9SD) for cases and 4.7(±2.1SD) years for controls.

Treatment was successful for the majority of patients. Most were rated as having good adherence (76% of cases and 93% of controls), and, at their last follow up visit, most had CD4 counts above 350 cells/ul (61% for cases, 69% for controls). In addition, the vast majority was at WHO stage 1 at their last follow up visit (99% of cases and 98% of controls) (Table 1).

Reflecting the fact that most patients started treatment with advanced disease, a significant proportion of the patients developed opportunistic infections (OIs) while under care (58% of cases and 34% of controls). The most common OIs were tuberculosis, which was experienced by 19% of patients, and which constituted 45% of OIs reported (see Table 2); bacterial pneumonia (13% of patients and 30% of reported OIs) and herpes simplex (12% of patients and 28% of reported OIs). Only 9 malignancies were reported: 3 cases of Kaposi’s sarcoma, 3 cases of cervical cancer, 1 case of colon cancer and 2 cases of unspecified malignancy. A small proportion of patients developed anemia (21% of cases and 11% of controls as defined by WHO’s standard for anemia among HIV-positive individuals) [37] (Table 2).

**Table 2:** Opportunistic infections among patients (*n* = 267)

Opportunistic Infections	Cases [no (%)]	Controls [no (%)]
Tuberculosis of all types	77 (62)	43 (30)
Bacterial pneumonia	29(23)	52(36)
Diarrheal disease of all type	13(10)	21(15)
*Pneumonitis Carni* Pneumonia	3(2)	5(3)
Herpes simplex virus	45(36)	54(38)

Sixty-five percent of cases and 26% of controls were recorded as having had a problem with eating during their care but only 10% of patients had received therapeutic food supplementation (28% of cases and 1% of controls). The most common reasons given for having an eating problem were loss of appetite (reported by 55% of those with eating problems), oral candidiasis (53% of patients), and nausea (33.33 of patients) (see Table 3).

**Table 3:** Reasons for eating problems (*n* = 429)

Causes of eating problems	Cases [no (%)]	Controls [no (%)]
Loss of appetite	97 (71)	40 (36)
Oral candidiasis	74 (54)	58 (52)
Esophageal candidiasis	18 (13)	8 (7)
Nausea and vomiting	39 (28)	44 (39)
Oral hairy leukoplakia	4 (3)	5 (4)

### Socio-economic support

Most patients reported having some source of social support for informal care-giving (91% of controls and 80% of cases) with spouses, priests and male siblings being the most frequently mentioned (see Table 4).

**Table 4:** Sources of informal care-giving support (*n* = 554)

Sources of Informal Care-giving Support	Cases [no (%)]	Controls [no (%)]
Parents	19 (11)	43 (11)
Religious father	29 (17)	54 (14)
Wife	18 (11)	73 (19)
Husband	32 (19)	77 (20)
Brother	21 (12)	41 (11)
Sister	30 (18)	53 (14)
Relatives	17 (10)	58 (15)
Child	28 (17)	42 (11)

More than half of the patients studied had disclosed their HIV status but there was a marked difference in HIV disclosure between cases and controls, with 61% of the cases having disclosed their status to someone but only 39% of controls having done so (see Table 5). Priests, wives, and children were the most common people to whom patients disclosed their status.

**Table 5:** Person to whom patients disclosed their HIV/AIDS status (*n* = 339)

To Whom Patients Disclosed	Cases [no (%)]	Controls [no (%)]
Parents	14 (17)	37 (14)
Religious father	14 (17)	77 (30)
Wife	15 (18)	53 (21)
Husband	8 (10)	46 (18)
Brother	12 (15)	27 (11)
Sister	12 (15)	20 (8)
Relatives	9 (11)	25 (10)
Child	23 (28)	50 (19)
Friend	2 (2)	17 (7)

### Determinants of undernutrition among patients

Table 6 summarizes the findings of our bivariate and multivariate analyses on the factors that associated with undernutrition among adult patients on ART. Older patients had slightly increased odds of developing under nutrition, with each additional year of age increasing the odds of undernutrition by 2% (AOR = 1.02, 95% CI: 1.01,1.05). Other standard demographic factors were not significantly associated with developing under nutrition in multivariate regression.

**Table 6:** Factors associated with undernutrition among Adult Patients receiving ART, Debre Markos referral hospital, Debre Markos Ethiopia

	Model 1: Bivariate	Model 2: Demographics	Model 3: Clinical Factors	Model 4: Social Support
Socio-Demographic Characteristics				
Male	0.903	0.816	0.792	0.870
	[0.64,1.27]	[0.57,1.17]	[0.53,1.19]	[0.57,1.32]
Age	**1.018** ^*^	1.023*	1.025*	**1.024** ^*^
	[1.00,1.04]	[1.00,1.04]	[1.00,1.05]	[1.01,1.05]
Has Children	0.853	**0.703^**	0.737	0.810
	[0.60,1.21]	[0.48,1.02]	[0.48,1.12]	[0.52,1.25]
Secondary Education or Higher	0.849	0.908	1.026	1.129
	[0.59,1.21]	[0.63,1.32]	[0.67,1.56]	[0.72,1.76]
Urban Residence	0.689	0.735	0.900	1.019
	[0.44,1.08]	[0.46,1.16]	[0.53,1.53]	[0.59,1.77]
Clinical Characteristics				
Experienced Opportunistic Infections	**2.769** ^***^		1.695**	**1.686** ^*^
	[1.97,3.89]		[1.14,2.51]	[1.12,2.52]
Experienced Anemia	2.052**		1.863*	**1.812** ^*^
	[1.31,3.21]		[1.11,3.13]	[1.07,3.07]
Experienced Eating Problems	**5.089** ^***^		3.813***	**3.400** ^***^
	[3.57,7.26]		[2.57,5.65]	[2.27,5.10]
Level of Adherence				
Good	0.240***		Base	Base
	[0.15,0.39]			
Fair	2.920***		2.792**	**2.774** ^**^
	[0.15,0.39]		[1.44,5.43]	[1.40,5.50]
Poor	5.611***		4.904^***^	**4.722** ^***^
	[2.55,12.4]		[2.04,11.8]	[1.92,11.6]
Baseline CD4 Count				
CD4 count of less than 200 cells/ul	1.953***		Base	Base
	[1.39,2.75]			
CD4 count between 200 and 350 cells/ul	0.637*		0.621*	**0.614** ^*^
	[0.43,0.94]		([0.40,0.97]	[0.39,0.96]
CD4 count greater than 350 cells/ul	0.548*		0.504*	**0.488** ^*^
	[0.33,0.90]		[0.28,0.89]	[0.27,0.88]
Socio-Economic Support				
Access to Public Services (housing, water, electricity)	**0.616** ^*^			1.062
	[0.42,0.90]			[0.67,1.69]
Has disclosed HIV status	**0.410** ^***^			**0.639** ^*^
	[0.29,0.57]			[0.43,0.95]
Has an informal caregiver	**0.398** ^***^			**0.475** ^**^
	[0.25,0.64]			[0.27,0.84]
Owns a phone	**0.530** ^***^			0.722
	[0.38,0.74]			[0.47,1.11]
Observations		636	636	635
*AIC*		810.8	694.9	684.1

Odds ratios reported; standard errors in parentheses

^^^*p* < 0.10, ^*^ *p* < 0.05, ^**^ *p* < 0.01, ^***^ *p* < 0.001

Patients’ clinical characteristics were consistent predictors of developing undernutrition. The factor most strongly and consistently associated with undernutrition was having poor adherence to treatment: holding other factors constant, those with poor adherence to treatment had 4.72 the odds of becoming malnourished than those with good adherence (AOR = 4.72, 95% CI: 1.92, 11.6) and those with fair adherence had more than double the odds (AOR = 2.77, 95% CI: 1.40, 5.50). The other clinical factor strongly associated with developing undernutrition was having experienced an eating problem while on treatment. The odds of becoming malnourished while on ART was 3.40 times higher for patients who had eating problems than those who did not after controlling for demographic factors, socio-economic support, adherence and for baseline severity of disease (AOR = 3.40, 95% CI: 2.27, 5.10). Having higher baseline CD4 counts reduced the odds of developing undernutrition (AOR = 0.61, 95% CI: 0.39, 0.96) for CD4 counts 200–350 cells/ul, and (AOR = 0.49, 95% CI: 0.27, 0.88) for CD4 counts > 350 cells/ul).

The presence of opportunistic infections was also significantly associated with undernutrition after controlling for other factors; patients who had developed OIs while on treatment had 70% higher odds of developing undernutrition, holding other factors constant, compared with those who had not had OIs (AOR = 1.70, 95% CI: 1.12, 2.52). Patients who developed anemia had 1.81 times the odds of developing undernutrition than those who did not (AOR = 1.81, 95% CI: 1.07, 3.07).

Turning towards social and economic support factors, we find that disclosure of HIV status and having a source of informal care-giving support reduced the odds of developing undernutrition (AOR = 0.64, 95% CI: 0.43, 0.95) and AOR = 0.48, 95% CI: 0.27, 0.84) respectively. Owning a phone halve the odds of developing undernutrition in bivariable regression. The significance and magnitude of the effect fades in multivariable analysis but approaches significance at the 10% level (AOR = 0.72, 95% CI: 0.47, 1.11).

We examined the data to see if our strongest associations were modified by gender or educational status. We found no evidence that the associations between undernutrition, eating problems, adherence, disclosure, or having a caregiver differed for women, the more highly educated, or urban dwellers (Table 6).

## Discussion

This study set out to examine the factors associated with adult ART patients developing under nutrition during the course of their treatment. We found that greater age, poor adherence, starting treatment with advanced disease and the presence of OIs, anemia, or eating problems, were all independently and positively associated with undernutrition. Having social support and having a source of informal care-giving, reduced the odds of undernutrition.

Many of these findings are unsurprising and in line with those recent studies on this topic. It is, for example, logical that the patients with OIs and eating problems would have dietary deficiencies and problems absorbing nutrients and that this, in turn, would lead to anemia and malnutrition. The link between low BMI and anemia among HIV-infected patients is well established [38] and Ethiopian studies conducted at Butajira and Humera hospitals [27, 39] found associations similar to ours. Studies conducted at University of Gondar and Butajira Hospital also found associations between eating problems and malnutrition among patients on ART [32, 39]. A positive relationship between the presence of OIs and undernutrition has also been found in studies of patients on ART in Ethiopia and Nepal [26, 27, 40, 41].

However, expected these results are, they underscore the importance of nutritional counseling focused on loss of appetite and nausea, close monitoring of anemia, and aggressive treatment of opportunistic infections, particularly those that can cause eating problems such as oral hairy leukoplakia, and oral and esophageal candidiasis. Of the OIs, tuberculosis was the most strongly associated with undernutrition and should be targeted for close monitoring.

Therapeutic feeding—“food by prescription”—has been shown to be effective at preventing and treating malnutrition in HIV-infected adults [42] and at increasing ART adherence [11, 26] but we were not able to assess its impact in this study as the number of patients receiving therapeutic feeding was very small, the sequencing of developing under nutrition and receiving food supplements difficult to ascertain from the data, and the therapeutic feeding variable highly correlated with other clinical characteristics. However, the (non-significant) trend in our data suggests that those receiving therapeutic food supplements may have better adherence when controlling for baseline clinical status, demographics and socio-economic support.

The strong association that we found between ART adherence and undernutrition is also fairly well established [3, 12, 15, 24] and in keeping with other recent studies in sub-Saharan Africa [43] that find a relationships between food insecurity, undernutrition, and difficulties in patient adherence and treatment initiation [14]. Several studies have found that concerns about having an adequate amount of food might people to stop or delay treatment [7, 15, 17]. In the Ethiopian setting, a case-control study in Tigray region found an association between adherence and getting enough food and food of adequate quality [13].

Research on the association between adherence and nutritional status, suggest a circular relationship, in which poor baseline nutritional status makes ART initiation and adherence more difficult and poor adherence, because it harms viral suppression, leads to under nutrition [13, 14]. In contrast, adequate dietary intake and absorption allows patients to fully benefit from ART [44]. In support of this argument, researchers have found that patients who begin ART without adequate nutrition have lower survival rates [45, 46]. Based on the results of studies such as these, researchers have argued that a balanced diet and proper nutrition improves the effectiveness of antiretroviral drugs, and may also decrease the severity of medication side effects and the complications of OIs. While the structure of our study does not allow us to determine the direction of the relationship between adherence and nutritional status, it does lend credence to these arguments.

In this study, having disclosed one’s HIV status and having someone to count on as an informal caregiver reduced the odds of becoming malnourished. Previous research has found that disclosure and family support are associated with better prevention and treatment outcomes [47–50] and with improvements in ART initiation and adherence [51] in a variety of settings. Patients who disclose their sero-status seem to be better able to access the care and support that they need to manage their illness well. The social support that can come with disclosure has also been associated with greater self-esteem and lower levels of depression [52].

When we look at disclosure and nutritional outcomes specifically, the association is not as well established. Most studies examining nutritional outcomes do not include disclosure as an explanatory variable; and the studies of nutritional outcomes in ART patients in Ethiopia that did include it find no association [53]. Given the established association between disclosure and other clinical outcomes, future studies should consider including it as an independent variable in explanatory models.

Our finding patients without a caregiver were two times more likely to develop undernutrition compared with patients with caregivers is also relatively novel as this variable is not included in most nutritional status. Even though research is limited on this topic, our findings are not surprising as it is intuitive that having an informal caregiver would help people with living HIV to adhere to treatment and to maintain their quality of life. Given the strong association found here, further study of the role that caregivers can play in nutritional counseling and support may be warranted. In addition, when we look at sources of support, we find that the “other” source, which we believe would most likely be a community volunteer or a friend, was the most strongly associated with good nutritional status. Similarly, disclosure to a friend was more strongly associated with good nutritional status than disclosure to family members or priests. This suggests that HIV groups and community-based care programs may be an important source of information and support and should continue to be developed.

A result that is surprising is the lack of association found between our nutritional outcomes, and our proxies for socio-economic status. We found no association between undernutrition, educational level, urban residence, home ownership and access to public municipal goods such as electricity and piped water. Wealth or income of patients on ART has been found to be negatively associated with undernutrition in Haiti [54], and Brazil [55], as was educational status in Senegal [19] and wealth in Tigray, Ethiopia [43]. The lack of association may reflect the largely urban make up of our sample or simply the weakness of these measures in capturing socio-economic status.

Finally, our study found that age and poor nutrition status were positively related. The evidence on age as a risk factor for undernutrition is mixed. It was found to have weak positive association with malnutrition in Brazil, no effect on low BMI in Haiti, and a negative association with malnutrition in Senegal. In older HIV-infected adults, more advanced age is associated with lower BMI [56] although the evidence is mixed [19, 54]. The uncertainty around the impact of age on nutritional status suggests that further study on this topic is needed.

### Limitations

This study has several limitations. First, it is a single-facility study that include only patients that are currently in care, which limits its generalizability. Second, it uses existing patient records its secondary data source, which limits the number of variables that we can include in our models. For example, we were not able to include information on household dietary diversity, baseline BMI and food security, or patient occupation, all of which might have had important associations with nutritional status. Most important, because our outcome variable measured whether the patient had ever developed undernutrition while on treatment, we were not able to determine the direction of relationships between some patient clinical characteristics such as adherence or having had an OI and developing undernutrition.

## Conclusions

Our findings suggest that ART clinics like those at Debre Markos Referral hospital’s and other similar settings of the country should develop tools and procedures for the early diagnosis of eating problems, anemia and opportunistic disease so that they can intervene in a timely manner during patient follow-up visits. Treatment should be started as early as possible with adherence support and close monitoring of patients for opportunistic infections that might affect eating and drug side effects that may affect appetite. The role of disclosure, peer-caregivers and age in preventing under nutrition should be explored in future research. Support group and community/home-based care programs should be explored for patients living with HIV should be encouraged as a means for encouraging disclosure, providing support, and sharing strategies for maintaining a good nutritional status.

## Abbreviations

AIDS: Acquired Immune Deficiency Syndrome
AOR: Adjusted Odds Ratio
ART: Anti-Retroviral Treatment
BMI: Body Mass Index
COR: Crude Odds Ratio
HIV: Human Immune Deficiency Virus
IDUs: injection drug users
SD: Standard Deviation
SPSS: Statistical Package for Social Sciences
WHO: World Health Organization
